# Modification of PCNA by ISG15 plays a crucial role in porcine deltacoronavirus infection

**DOI:** 10.1186/s13567-026-01802-1

**Published:** 2026-07-11

**Authors:** Cheng Li, Xiao-ran Guo, Yu-jin Gao, Ling-zhai Meng, Hong-qing Zheng, Xiu-li Li, Zhi-min Dong, Wei-ke Ren, Hui-zhong Sun, Fu-qiang Li, Qi Zhu, Linyuan Feng, Li Zhang, Ming-hua Yan

**Affiliations:** 1 Institute of Animal Science and Veterinary, Tianjin Academy of Agricultural Sciences, Tianjin, 300381 China; 2 Tianjin Key Laboratory of Animal Molecular Breeding and Biotechnology and Tianjin Engineering Research Center of Animal Healthy Farming, Tianjin, 300381 China; 3 Key Laboratory of Animal Epidemic Disease Diagnostic Laboratory of Molecular Biology in Xianyang City, Polytechnic Institute, Xianyang Vocational Technical College, Xianyang, 712000 Shaanxi China; 4 College of Animal Science and Veterinary Medicine, Tianjin Agricultural University, Tianjin, 300384 China; 5 Institute of Agro-Product Safety and Nutrition, Tianjin Academy of Agricultural Sciences, Tianjin, 300381 China; 6 Beijing Professional Business Institute, Beijing, 100010 China

**Keywords:** Porcine deltacoronavirus, ISG15, innate immunity

## Abstract

Porcine deltacoronavirus (PDCoV), an emerging enteropathogenic coronavirus belonging to the deltacoronavirus genus, has caused massive losses to the livestock industry and poses a potential risk of cross-species transmission. Interferon-stimulated gene 15 (ISG15) plays a crucial role in virus infection and immune regulation through interactions with cellular and viral proteins. However, the mechanisms underlying the effect of ISG15 on PDCoV infection are poorly understood. In this study, we demonstrated that the knockout of ISG15, mediated by CRISPR-Cas9, facilitates PDCoV replication and that ISG15 modification (ISGylation) deficiency attenuates the antiviral ability of ISG15. To clarify the novel roles of ISG15 during PDCoV infection, we constructed a stable ISG15-overexpressed IPEC-J2 cell line. We employed tandem affinity purification followed by mass spectrometry analyses with ISG15, enabling the identification of 538 known and novel candidate ISGylation proteins including proliferating cell nuclear antigen (PCNA) which is a sign of DNA damage. We showed that ISGylation of PCNA significantly inhibited PDCoV replication. During PDCoV infection, HERC5, an ISG15 E3 ligase, interacted with PCNA and promoted its ISGylation, whereas USP18 eventually de-ISGylated PCNA. Our results not only reveal that ISGylation acts as a post-translational modification of PCNA but also elucidate the function of ISGylation in PCNA during PDCoV infection, thereby adding a novel layer of complexity to the virus–host interaction interface.

**Supplementary Information:**

The online version contains supplementary material available at https://doi.org/10.1186/s13567-026-01802-1.

## Introduction

Coronaviruses (CoVs) are positive single-stranded RNA viruses with large genomes and are known to cause respiratory and gastrointestinal diseases in humans and animals [1]. The porcine deltacoronavirus (PDCoV) belongs to the Deltacoronavirus genus. It is notable that the PDCoV has its origins in Hong Kong, where it was first reported in 2012. Following this, the virus was characterized in the United States in 2014. Clinically, PDCoV has been observed to cause severe diarrhea, vomiting, and dehydration in nursing piglets, sharing characteristics with other swine enteric CoVs [2]. Co-infections of PDCoV with other swine enteric CoVs are frequently reported on pig farms, resulting in substantial economic losses for the livestock industry. Furthermore, it has been determined that there are potential risks associated with cross-species transmission and public health security in relation to PDCoV infection [3].

The effective replication of PDCoV in host cells and the antagonism of host immune response involve a variety of important signal transduction pathways in cells, which is a complex biological process. In this process, post-translational modifications can shape the strength and duration of signal transduction, such as those involving ubiquitin and other types of ubiquitin modification. Among them, interferon-stimulated gene 15 (ISG15) modification (ISGylation) plays an extremely important role in regulating the infection and replication of pathogenic microorganisms and the innate immune response of host cells. ISG15, a protein induced by interferon with a molecular weight of approximately 15 kDa, is composed of two small ubiquitin-like (Ubl) subunits connected by a short hinge sequence. ISG15 conjugates to modify proteins by a three-step enzymatic cascade including E1 enzyme Ube1L, E2 enzyme UbcH8, and E3 ligases (HERC5, HERC6, EFP and ARIH1); this process is ISGylation [4–6]. ISGylation is a reversible covalent modification that regulates the stability, functional activity, and intracellular localization of substrate proteins [7]. Meanwhile, to avoid over-activation of ISGylation, de-ISGylases such as USP18 or USP10 are expressed by the host to remove ISG15 from specific targets [6, 8].

Unlike ubiquitin, ISG15 and the ISGylation machinery are robustly induced by type I IFN and can be affected by viral infection. A fine-tuned response to environmental signals is necessary for the anti-viral potency of IRF3. ISG15, along with HERC5, modifies the anti-viral action of IRF3 by ISGylating IRF3 at three residues, leading IRF3 to fail to be targeted by the RING E3 ligase Pin1 for K48 polyubiquitination [9]. In addition to the innate immune factor STING protein, E3 ISGylation ligase proteins facilitate STING activation by ISGylating STING, inhibiting its K48-linked degradation [10]. HCMV pUL26 protein, as a substrate of ISGylation, inhibits the increase of ISGylation levels caused by HCMV infection [11]. HERC5 can catalyze the binding of ISG15 to IAV NS1 protein, thereby preventing NS1 protein from forming homodimers and inhibiting viral replication [12]. For CoVs, de-ISGylation of ISG15 appears to be a strategy that has evolved to antagonize the anti-viral effect of ISG15. SARS-CoV-2 can antagonize the activation of MDA5 by ISGylation during infection to achieve persistent infection and can unbind IRF3 to ISG15 by ubiquitination, thereby attenuating the IFN-I response [13]. It has been suggested that PDCoV PLpro can deubiquitinate and de-ISGylate by hydrolyzing ISG15-AMC substrates [14]. It is evident that the concurrent evolution of the virus, which has developed strategies to evade the immune system, is explained by these virus-adapted immune-escape strategies, which in turn antagonise the ISGylation machinery [15]. Therefore, identifying specific cellular host targets modified by ISG15 during PDCoV infection is crucial for understanding the molecular mechanism of host resistance to viral infection mediated by ISG15.

Advances in LC–MS/MS have yielded hundreds of new ISGylation targets, many of which have been confirmed by other methods. In this study, we overexpressed tagged ISG15 using lentivirus-mediated transfection to isolate ISG15 conjugates for analysis. We found that PCNA specifically ISGylated. It has been established that PCNA is an essential component in processes related to both the repair and bypass of DNA damage. In addition, PCNA functions as an organisational hub, coordinating the recruitment of vital components that form part of the DNA damage response [16]. PCNA enhances HBV and SARS-CoV-2 replication[17, 18]. In the context of PRRSV infection, the redistribution of PCNA from the nucleus to the cytoplasm has been observed. This redistribution has been implicated in the promotion of PRRSV replication through the augmentation of viral genome synthesis [19]. Despite the endeavours undertaken to elucidate the mechanisms by which ISG15 protects host cells during infection, the intricacies of the ISG15-PCNA regulatory axis remain to be fully delineated. Our results add a novel layer of complexity to the virus–host interaction interface and reveal how ISGylation of PCNA, catalyzed by HERC5 and de-ISGylated by USP18, could play a crucial role in tipping the balance of infection toward benefiting PDCoV infection.

## Materials and methods

### Cell culture and virus

HEK293T cells and IPEC-J2 cells were obtained from American Type Culture Collection and grown in DMEM, supplemented with 10% FBS at 37 ℃ in a 5% CO_2_ atmosphere. PDCoV (Genbank No: PZ291609) which previously isolated from small intestine specimens of piglets was stored in −80 °C ULT freezer in our laboratory [20]. PDCoV was incubated in DMEM supplemented with 10 mg/mL trypsin and used to infect IPEC-J2 cells. After adsorption for 1 h, a serum-free DMEM medium containing trypsin was changed into the wells.

### Overexpression and knockout of ISG15 in PDCoV permissive cells

The gene encoding the porcine ISG15 protein was fused with the Flag and His, and subsequently cloned into a CMV- vector. This process resulted in the generation of a vector designated as CMV-ISG15, which was found to express the EGFP protein. The primers used in this study are listed in Table 1. CMV-puro lentiviral vector (CMV) was generally used as a control. The sequence of swine ISG15 (EU647216.1) was found in the NCBI, and one specific site was selected as the sgRNA target sequence by software analysis. The sgRNA target sequences aare listed in Table 1. The sgRNA was inserted into the pRP[2CRISPR]-U6-BbsI-CBh-hSpCas9-CMV > Puro vector to obtain recombinant plasmid pRP[2CRISPR]-sgRNA. The mixed recombinant and auxiliary helper vectors mixed were transferred into HEK293T cells. Lentivirus carrying Flag-His-ISG15 or sgRNA was packaged and transduced into IPEC-J2 cells at MOI = 1. Monoclonal IPEC-J2 cell lines were cultivated with puromycin at a concentration of 1 μg/mL.

**Table 1:** **Primers used in this study**

Primers	Sequence (5′–3′)	Purpose
CMV-ISG15-F	ATCGCGTCTCGGGCTGCCACCATGGTGAGCAAGGGCGAGGAGGA	Amplification of ISG15
CMV-ISG15-R	ATCGCGTCTCGGGGTTTACTTGTACAGCTCGTCCATGCC	
pCAGGS-ISG15-F	CATCATTTTGGCAAAG ATGGGTAGGGAACTGAAGGTGA	Amplification of ISG15
pCAGGS-ISG15-R	CGAGAGATCTGAATT TCA CTTATCGTCGTCATCCTTGTAATC GCACTCGGTGGGGTGCTCCCCT	
PDCoV-M-F PDCoV-M-R	ATTCTGCTTTGGCTGCTC TCCTGTGGCGGATTTC	RT-qPCR for detection of PDCoV
GAPDH-F GAPDH-R	CTGCCGCCTGGAGAAACCT GCTGTAGCCAAATTCATTGTCG	RT-qPCR for detection of GAPDH
IFITM3-F IFITM3-R	TCAACATCCGAAGCGAGACC GAGTAGGCGAAAGCCACGAA	RT-qPCR for detection of IFITM3
BST-2-F BST-2-R	GTGGTGGGTCTGCTGGTGTC CAGATGGTCCGTTTGGTTTTG	RT-qPCR for detection of BST-2
ISG15-F ISG15-R	AGGGAACTGAAGGTGAAGATG CAGACGCTGCTGGAAGG	RT-qPCR for detection of ISG15
Mx1-F	TCTGTAAGCAGGAGACCATCAACT	RT-qPCR for detection
Mx1-R	TTTCTCGCCACGTCCACTATC	of Mx1
sgRNA	GCAGCAACGCCTATGAGGTC	Target ISG15 sgRNA

### RNA extraction and qPCR

The isolation of total RNA from IPEC-J2 cell was accomplished by employing the TRIzol reagent (Thermo Fisher Scientific), in accordance with the manufacturer's guidelines. IFITM3, ISG15, BST-2 and Mx1 were determined by qPCR. SYBR Premix Ex Taq™ (Takara Bio) was utilised according to the manufacturer's protocol, and specific primers that had been previously described were employed [21].

### TCID_50_ assay

A quantity of 100 μL/well was added to the IPEC-J2 cells, which were cultivated in the 96-well cell culture plates. The inoculation was conducted in accordance with the tenfold serial dilution of PDCoV. The plates were subsequently allocated a further eight replicates for each dilution. Following a one-hour adsorption process, DMEM medium containing trypsin was introduced into the well, which was then left to stand for a period of three days. The collection of the aforementioned supernatants was followed by the subsequent calculation of PDCoV titers as TCID_50_, in accordance with established protocols and using the Reed-Muench method.

### Western blotting

Cell were harvested and lysed with RIPA buffer (Thermo Fisher Scientific). Nuclear and cytoplasmic proteins of IPEC-J2 were isolated using an ExKine™ Nuclear and Cytoplasmic Protein Extraction Kit (Abbkine). The concentrations of the protein samples were quantified using a Protein Assay kit (Beyotime), and the samples were subsequently detected as previously described [8].

### Identification of novel ISG15-interacting proteins using TAP-LC–MS/MS in ISG15-overexpressing IPEC-J2 cells

In order to identify potential proteins that were physically associated with or covalently conjugated to ISG15, an ISG15-IPEC-J2 cell line was established (Figure 6A). This cell line was generated using a 6 × His-tag and Flag-tag-fused ISG15 protein and then analyzed using TAP-LC–MS/MS as described previously [21].

### In vitro ISGylation assays

IPEC-J2 cells were plated in six-well plates one day before transfection. Cells were co-transfected with pCAGGS-UbE1L (E1), pCAGGS-UbcH8 (E2), pCAGGS -HERC5 (E3), and Flag-ISG15 or Flag-ISG15AA. At 24 h post-transfection, cells were harvested for western blotting analysis with anti-ISG15 antibodies.

### co‑IP assays

IPEC-J2 cells were co-transfected with of plasmids (2 μg). Co-transfected cells using TurboFect (Thermo Fisher Scientific) were cultured for 48 h and lysed on ice. The monoclonal antibody to HA, Myc, or Flag was mixed with the cell liquid for 8 h. Beads were incubated at 4 ℃ overnight. The immunoprecipitate was analyzed with anti-HA or -ISG15 antibodies.

### Pulldown assay

Plasmids expressing UBE1L (E1), UbcH8 (E2), and Flag-ISG15 were cotransfected into IPEC-J2 cells. The cells were exposed to NEM (5 mM) for a duration of 30 min prior to being harvested. Cell lysates were prepared at 48 h after transfection. HisMax-PCNA or HisMax-PCNA-K168 was pulled down using Ni–NTA resins. Following preliminary purification, the purified cell extracts were left to react with Ni–NTA resins for 16 h at 4 °C. We prepared ISGylated proteins from co-transfected cells and added them to HisMax-PCNA or HisMax-PCNA-K168. The mixture was subjected to several washes with co-IP buffer after an incubation period of 2 h at 4 °C. The samples were then analyzed by immunoblotting with the appropriate antibodies.

### Statistical analysis

The data are displayed as the mean ± standard error of three independent experiments per test. In all cases, a two-tailed unpaired t-test was used for comparisons between experimental groups. Tukey's post-hoc test was used to analyse the data, which was collected from multiple groups. The following symbols are used to denote significant differences: *p < 0.05, **p < 0.01,***p < 0.001. Three biological replicates were used for each condition.

## Results

### Porcine ISG15 protein expression is upregulated in response to PDCoV infection

**Figure 1:**
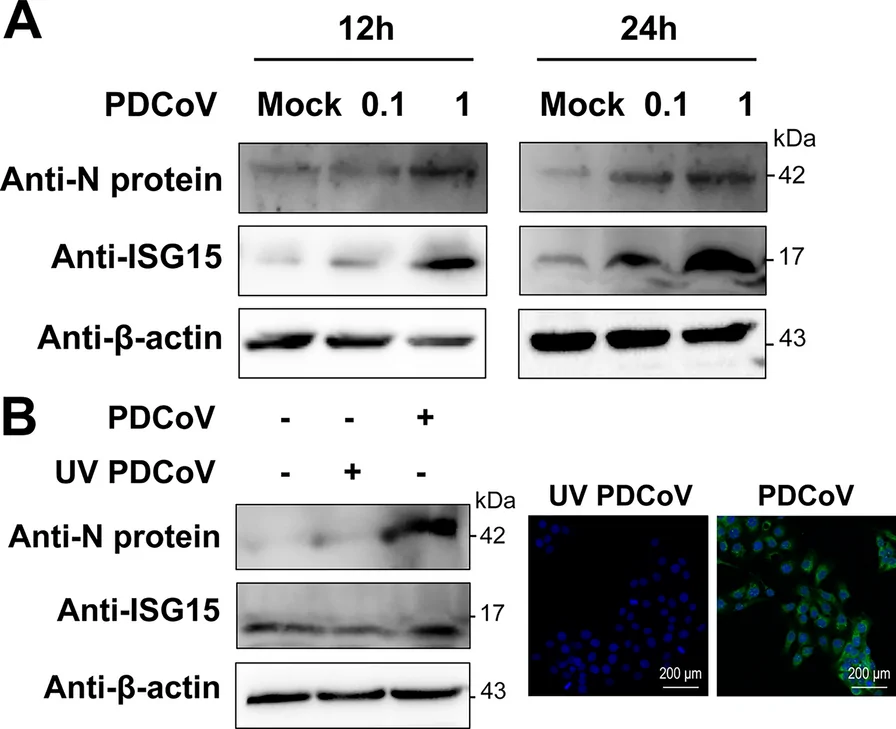
**Expressions of ISG15 along with PDCoV N protein were probed by western blotting. A** IPEC-J2 cells incubated with PDCoV for 12 h or 24 h. **B** PDCoV was inactivated by ultraviolet irradiation for 60 min. IPEC-J2 cells incubated with PDCoV and UV PDCoV (MOI = 0.1).

To verify the effect of PDCoV infection on ISG15 and ISGyaltion, IPEC-J2 cells were incubated with PDCoV. Following infection with varying doses of PDCoV, ISG15 protein expression was upregulated at both 12 and 24 hpi (Figure 1A). To determine whether the upregulation of ISG15 depends on virus replication, PDCoV was inactivated by ultraviolet irradiation, and the complete inactivation of the virus was detected (as shown in Figure 1B). IPEC-J2 cells infected with inactivated virus showed that PDCoV inactivation failed to induce ISG15 upregulation, indicating that ISG15 upregulation induced by viral infection was dependent on PDCoV replication.

### ISG15 expression in porcine cell lines

**Figure 2:**
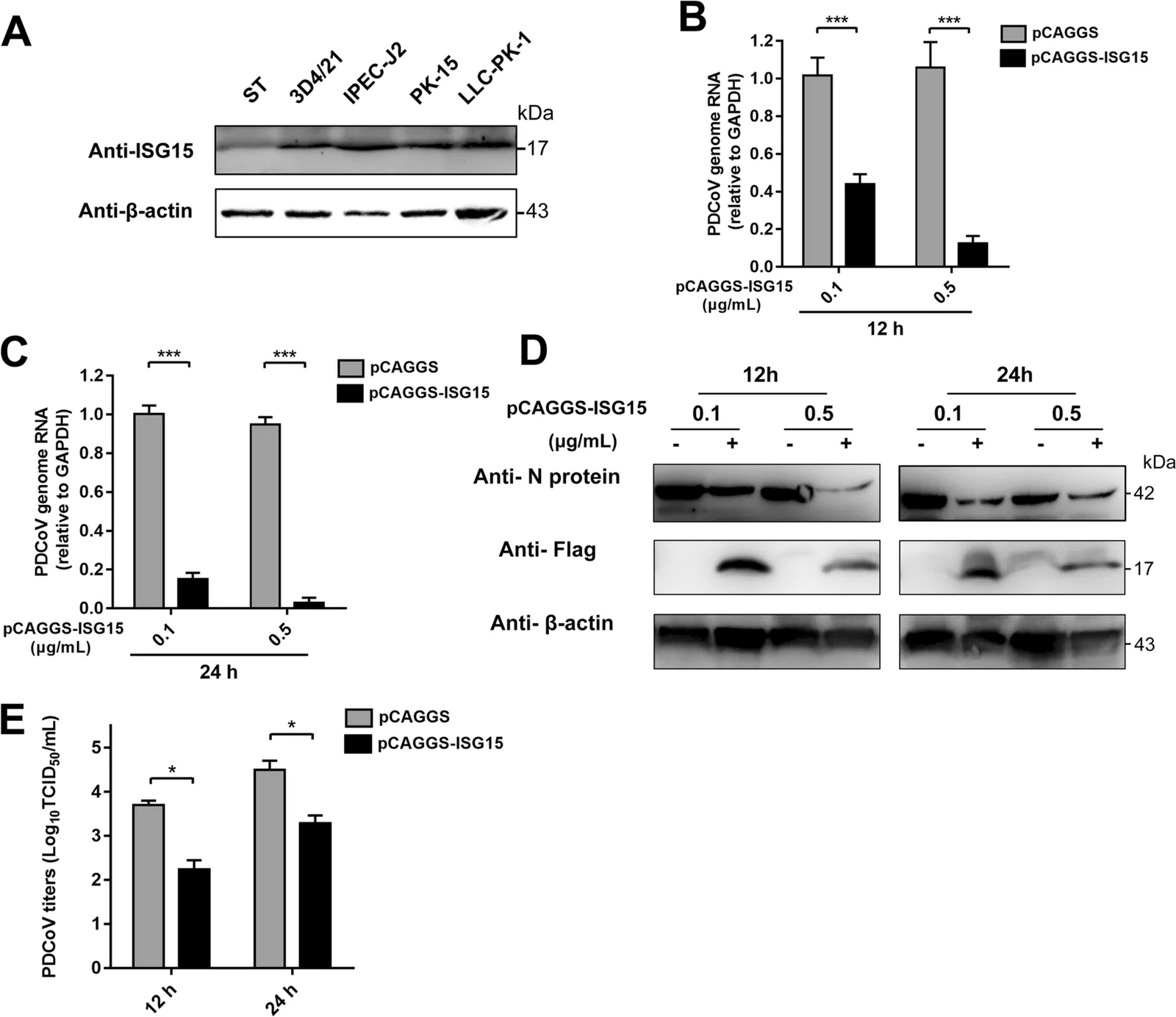
**PDCoV proliferation is inhibited by ISG15.** **A** Porcine ISG15 expression in swine cells. **B**–**D** PDCoV proliferation is inhibited by ISG15 in IPEC-J2s. IPEC-J2s transiently infected with pCAGGS-ISG15 in different doses (0.1 and 0.5 µg/µL). PDCoV M gene mRNA was analysed by RT-qPCR. PDCoV N protein and ISG15 were analysed by western blotting. **E** Viral titers in the supernatant are assessed and expressed as TCID_50_/mL.

The confirmation of the expression of swine ISG15 in various porcine cell lines, including Swine Testis cell line (ST), Porcine Alveolar Macrophage cell line (3D4/21), Intestinal Porcine Epithelial cell line (IPEC-J2), Porcine Kidney-15 cell line (PK-15) and Porcine Kidney 1 cell line (LLC-PK-1), was achieved through the utilization of a western blotting analysis. This analysis was conducted with the objective of ascertaining the protein expression level of porcine ISG15. As illustrated in Figure. 2A, porcine ISG15 demonstrates significant levels of expression in diverse swine tissues, including the testicles, lungs, intestines and kidneys, indicating a crucial, well-conserved function within these tissues.

### PDCoV proliferation is inhibited by ISG15

Swine ISG15 with the Flag-tag was cloned into a pCAGGS vector to generate pCAGGS-ISG15. Primers were listed in Table 1. To clarify the role of porcine ISG15 in PDCoV infection, different doses of pCAGGS-ISG15 or pCAGGS vector were transiently transfected into IPEC-J2s. Following transfection, IPEC-J2s were infected with PDCoV. PDCoV mRNA and PDCoV titers were determined by RT-qPCR and TCID_50_ assays, respectively. PDCoV N and ISG15 protein levels were tested by western blotting during this process. Compared to the mRNA levels of PDCoV in pCAGGS-transfected IPEC-J2 cells, those in pCAGGS-ISG15-transfected IPEC-J2s were inhibited at 12 and 24 hpi (Figure 2B–D). The viral titer of PDCoV in culture supernatants was distinctly inhibited following transfection with pCAGGS-ISG15 at 12 and 24 hpi (Figure 2E). These data indicated that pCAGGS-ISG15 overexpression decreased PDCoV multiplication in IPEC-J2 cells.

### Knockout of ISG15 mediated by CRISPR-Cas9 enhances PDCoV replication

**Figure 3:**
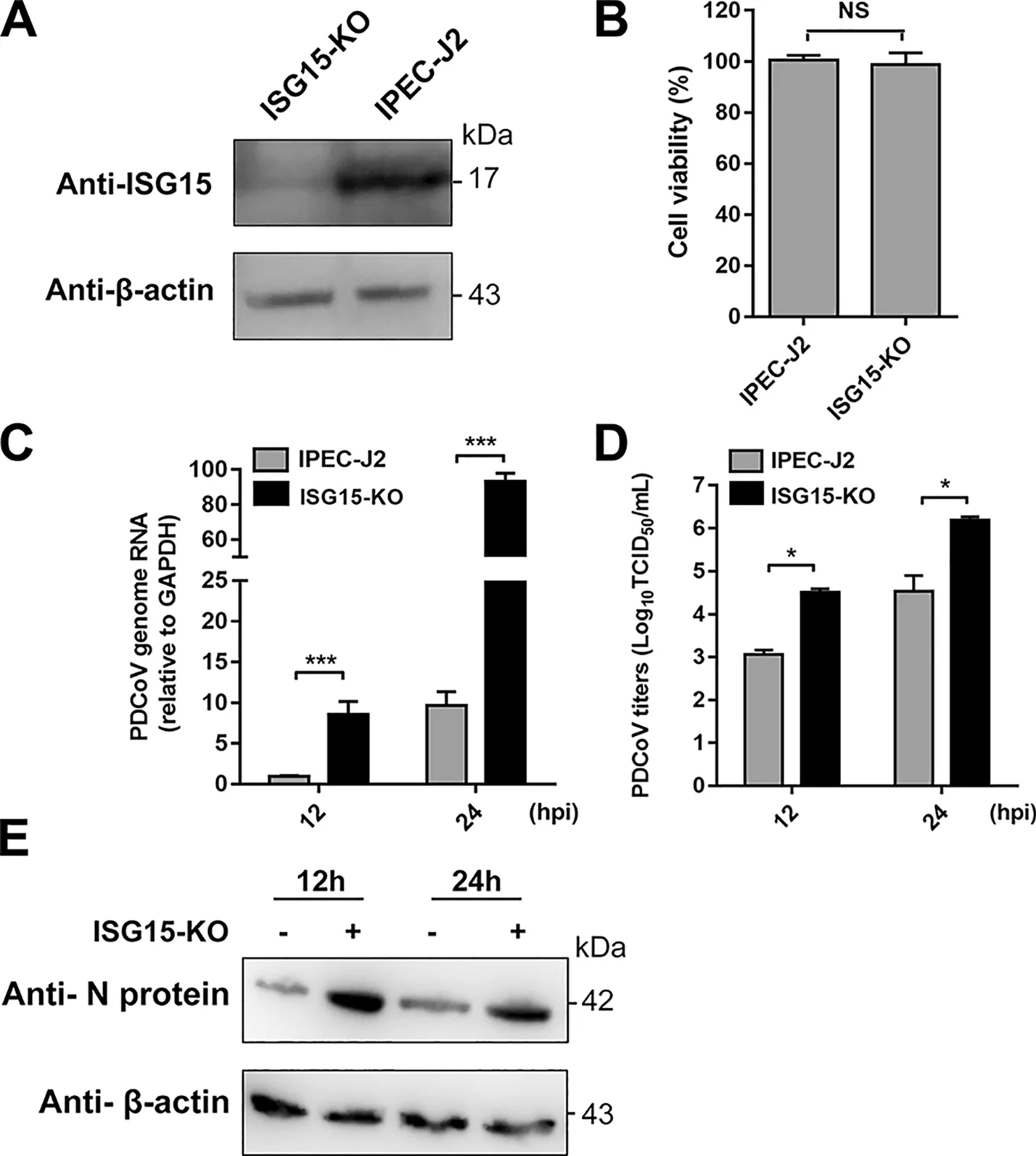
**Knockout of ISG15 promoted PDCoV replication.** Establishment of IPEC-J2-ISG15-KO cell. **A** Expression levels of ISG15. **B** Cell viability of ISG15 knockout cell lines. **C** PDCoV M gene mRNA was analysed by RT-qPCR. **D** Viral titers in the supernatant are assessed and expressed as TCID_50_/mL. **E** PDCoV N protein and ISG15 were analysed by western blotting.

To directly explore the role of ISG15 upon PDCoV infection, IPEC-J2 was used to construct an ISG15 knockout (IPEC-J2-ISG15-KO) cell line via CRISPR-Cas9 technology [22], which normally expresses a high level of swine ISG15 following IFN stimulation [8]. We isolated a clone that exhibited a loss of ISG15 expression. As shown in Figure 3A, ISG15 protein was not visible in IPEC-J2-ISG15-KO cells by western blotting with ISG15 antibody, proving that the establishment of the knockout cell lines was successful. The propagation and viability of cell lines did not significantly vary (Figure 3B). IPEC-J2-ISG15-KO were assessed for the effect of ISG15 knockout on the virus at 12 or 24 hpi with PDCoV (MOI = 0.1). The viral mRNA and N protein of PDCoV in ISG15 knockout cells were higher than those in the control group at 12 and 24 hpi (Figure 3C and E). Concomitantly, PDCoV titers at 12 and 24 hpi were increased in IPEC-J2-ISG15-KO cells compared to IPEC-J2s (Figure 3D).

### ISGylation participates in the anti-viral activity of ISG15 against PDCoV

**Figure 4:**
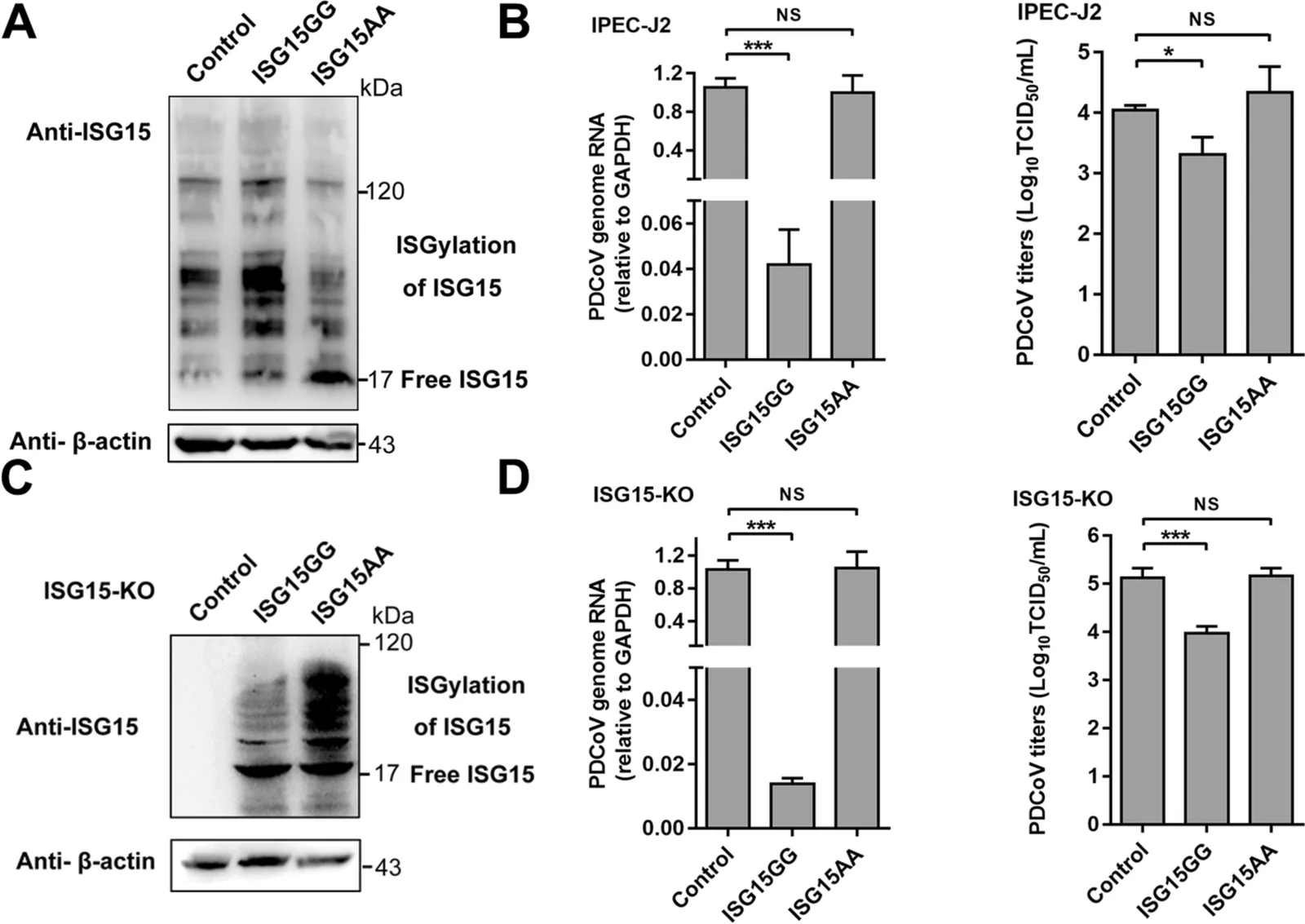
**IPECs or IPEC-J2-ISG15-KO cells were transfected with ISG15GG or ISG15AA.** **A** and **C** ISGylated protein was detected by western blotting. IPEC-J2s (**B**) and ISG15-KO IPEC-J2s (**D**) were transfected with ISG15GG or ISG15AA followed by PDCoV infection for another 24 h (MOI = 0.1). PDCoV replication was quantified by RT-qPCR and viral titer analyse.

To determine if ISG15 functions as an anti-PDCoV agent through a conjugated mechanism, site-directed mutagenesis was used to construct a mutated form of ISG15 whose C-terminal LRLRGG motif was mutated to LRLRAA motif [8]. Only free ISG15 was increased in pCAGGS-ISG15AA-transfected IPEC-J2 cells and compared to that in IPEC-J2 cells, while the level of ISGylation seen in the ISG15AA-transfected cells was not as high as that seen in the pCAGGS-ISG15GG-transfected cells. This suggests that the ability of the mutated form of ISG15 (GG to AA) to conjugate with the target protein has been lost. (Figure 4A). IPEC-J2s with the pCAGGS plasmid transfection were used as control. PDCoV mRNA and viral titers in pCAGGS-ISG15GG or pCAGGS-ISG15AA-transfected IPEC-J2s were tested by RT-qPCR and TCID_50_/mL assay (Figure 4B). The results show that key site mutations resulted in a significant reduction in the inhibitory effect of ISG15 against PDCoV. This suggests that the inhibition of PDCoV proliferation by ISG15 is dependent on ISGylation. Similar experiments were performed in IPEC-J2-ISG15-KO cells. These yielded similar results (Figure 4C and D). These cells were used to exclude the impact of basal ISG15 levels. The results revealed that conjugative ISG15 can inhibit PDCoV.

### Identification of His-Flag-tagged ISG15 modified protein expression in IPEC-J2s

**Figure 5:**
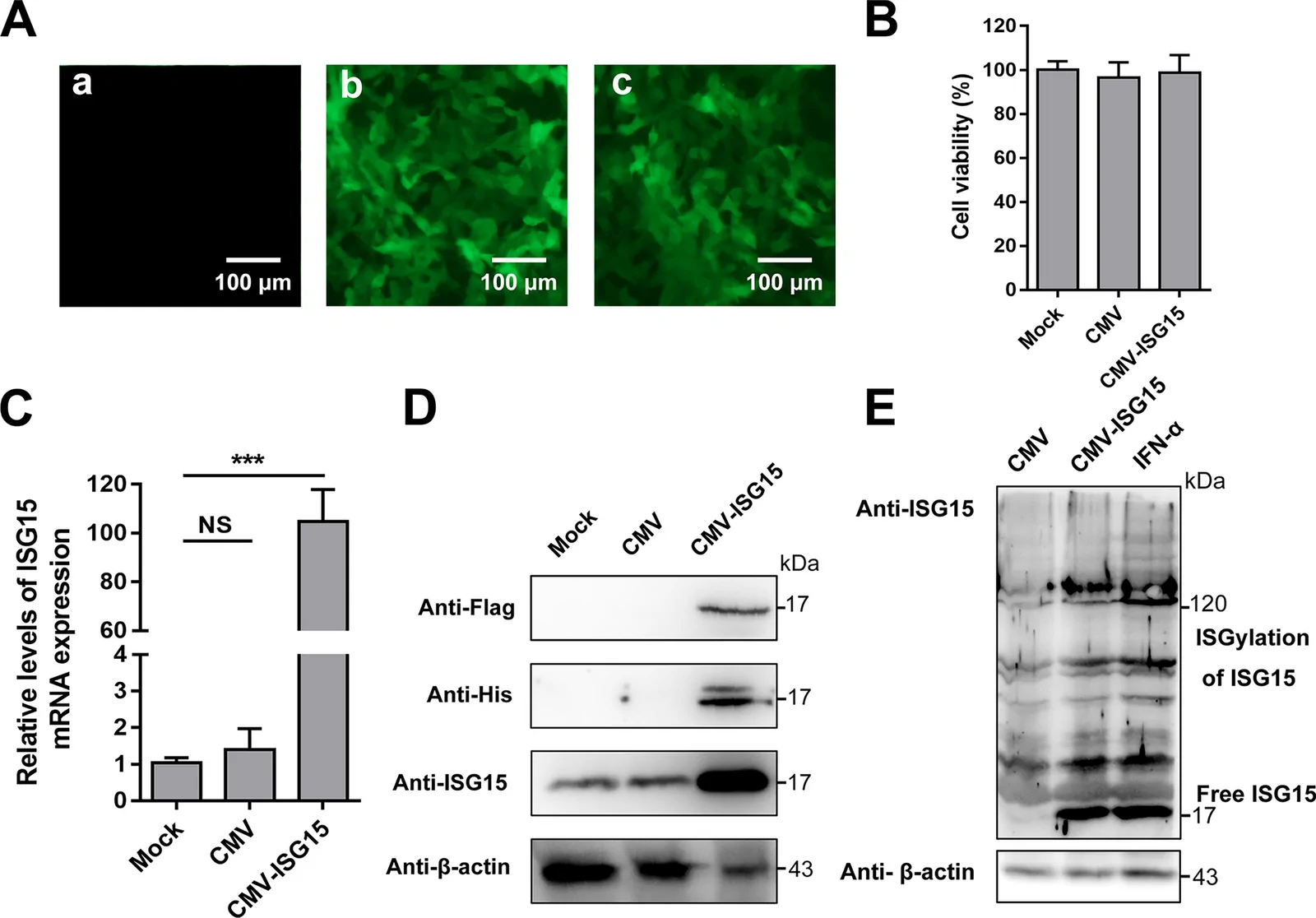
**The stable expression of ISG15 in IPEC-J2s was achieved through the use of lentiviral transduction.** **A** EGFP reporter expression was detected in mock (**a**), CMV (**b**), and CMV-ISG15-transfected IPEC-J2 cells (**c**) under fluorescence microscopy. Scale bars: 100 μm. **B** Cell vitality of CMV-ISG15-transfected cells was measured. **C**–**E** ISG15 mRNA and protein in CMV- and CMV-ISG15-infected cells was analysed by RT-qPCR and western blotting.

To develop a comprehensive understanding of how ISG15 plays an anti-viral role during the course of PDCoV infection, we established an overexpression IPEC-J2s cell line via lentivirus packaging technology, which expressed a His- Flag-ISG15 fusion protein (CMV-ISG15). IPEC-J2s transfected with CMV plasmid was treated as a control. EGFP was visual in CMV-ISG15 and CMV cells (Figure 5A). The viability of the cells was not significantly affected (Figure 5B). The mRNA expression of ISG15 was upregulated considerably in CMV-ISG15 (Figure 5C). His-Flag-ISG15 expression was detected at approximately 19 kDa, respectively (Figure 5D). The CMV-ISG15 protein level was also tested by anti-ISG15 antibody and was particularly increased in CMV-ISG15 cells. Furthermore, free ISG15 protein levels and ISGylation levels were determined in ISG15-overexpressing cell lines or following IFN-α 100 μg/mL pretreatment for 12 h (Figure 5E). Only overexpressed ISG15 induced ISGylation without co-translating with another ISGylation enzyme or IFN pretreatment. Thus, CMV-ISG15 cells can be used to screen ISGylated proteins in subsequent experiments.

### Identification of novel ISG15-interacting proteins using TAP-LC–MS/MS in ISG15-overexpressing IPEC-J2 cells

**Figure 6:**
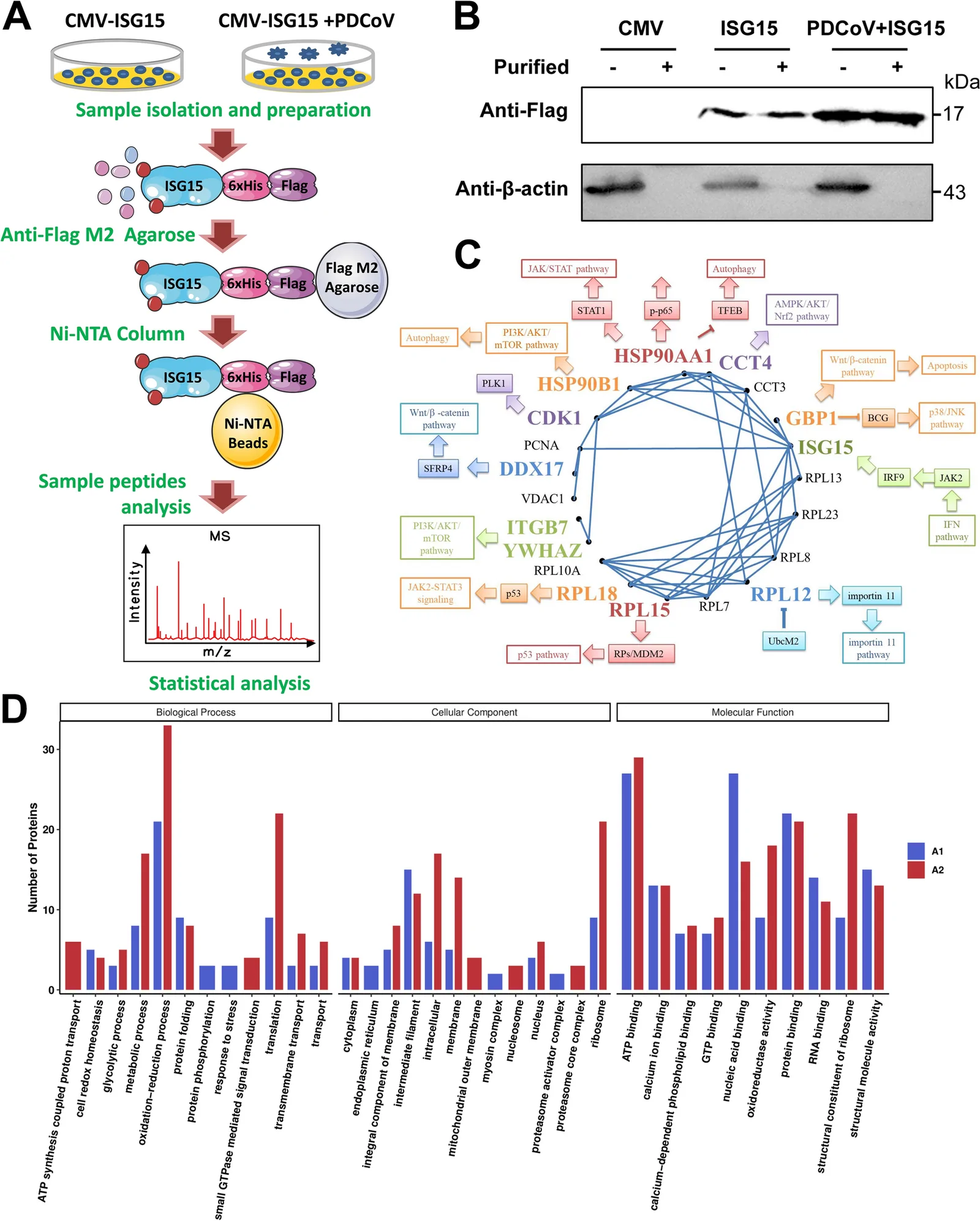
**Proteins associated with and/or modified by ISG15 are to be identified and purified.** **A** The Flag-His-TAP procedure is shown schematically. **B** Verification of the ISG15 tandem affinity purification process by means of western blotting with the anti-Flag antibody. **C** Functional interaction map for ISG15. **D** Gene Ontology analysis of the identified ISG15-interacting proteins categorized according to biological process, cellar component and molecular function.

We conducted affinity pulldown experiments using total PDCoV-infected CMV-ISG15 cell lysates and fusion ISG15 protein. The uninfected CMV-ISG15 IPEC-J2 cells were used as controls (Figure 6A). The purified protein complexes from cells infected with PDCoV or uninfected cells were detected (Figure 6B). SDS-PAGE was performed to monitor the quality of the two-step purification, visualized using the Coomassie brilliant blue dye method (Additional file 1C). After excluding the 185 proteins jointly identified in two groups, 141 proteins identified in uninfected cells and 212 proteins in PDCoV-infected cells that may interact with ISG15 were identified (Additional file 1A). The Venn diagrams of the IPR, GO, KEGG, and COG databases that annotated the results are shown in Additional file 1B. We short-listed a set of 30 ISG15 conjugated proteins, three of which have been previously identified as ISG15 interactors, namely PCNA, IQGAP1, and filamin-B isoform 2 [23]. The other ISG15-associated proteins included GBP1, CDK1, and integrin beta (Table 2). Several of the identified ISG15 interaction partners were RPL family members (RPL7, RPL8, RPL10A, RPL12, RPL13, RPL15, RPL18 and RPL23) responding to changes in growth conditions and cellular status [24]. Subsequently, proteins identified from ISG15-interacting proteins were selected to establish an interaction networks using IPA. According to their scores, 20 proteins were chosen and constructed into an diagram, as shown in Figure 6C. To illustrate the functional association of ISG15-interacting proteins, GO analysis was performed (Figure 6D). The oxidation–reduction process and translation were the most represented GO terms, which were obviously increased in the PDCoV-infected group. A number of crucial pathways were identified as being enriched in the interacting proteins from the PDCoV-infected group (Additional file 1D). A number of protein domains were significantly enriched in the PDCoV-infected group, including the RNA recognition motif domain, intermediate filament protein domain, and chaperonin Cpn60/TCP-1 family domain (Additional file 1E). The RNA recognition motif domain is important for binding various RNA sequences and proteins [25].

**Table 2:** **Antibodies used in this study**

Antibody	Manufacturer	Catalog No
Anti-β-actin	Abcam, Cambridge, UK	mAbcam 8226
anti-ISG15	Proteintech, Wuhan, China	15981-1-AP
anti-Flag	Cusabio, Wuhan, China	CSB-MA000021M0m
anti-Myc	Sigma, St. Louis, MO, USA	M4439
anti-6X His tag	Abcam, Cambridge, UK	EPR20547
anti-HA	Sigma, St. Louis, MO, USA	H3663
anti-HERC5	Proteintech, Wuhan, China	22692-1-AP
anti-N	prepared and stored in our laboratory	[21]

### ISG15 suppress PDCoV replication via conjugate to PCNA

**Figure 7:**
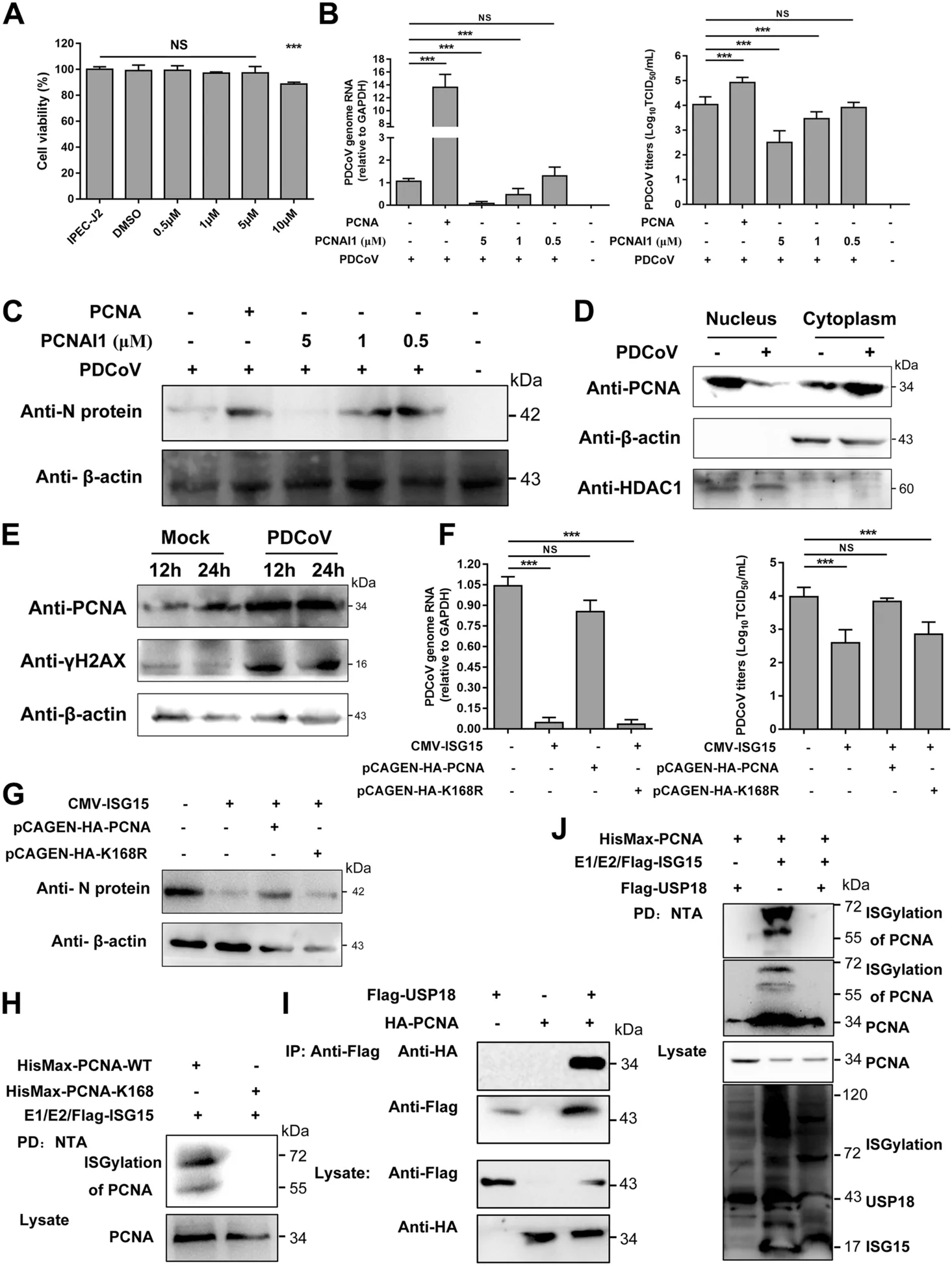
**Effects of PCNA on PDCoV infection.** **A** The CCK-8 assay was used to determine the safe concentration of PCNA-I1. **B** and **C** IPEC-J2s were infected for 12 h and then performed with different amounts of PCNA-I1. IPEC-J2s transfected with PCNA-overexpressed plasmid were served as positive control. At 24 h, the inhibitory effect of PCNA-I1 on PDCoV was analysed by RT-qPCR and western blotting. Viral titers are assessed as TCID_50_/mL. **D** IPEC-J2s were infected with PDCoV. After a 48-h period, extraction was carried out on both the nuclear and cytoplasmic components. The expression levels of PCNA were then analysed through the implementation of a western blotting assay. β-actin and HADC1 were used as controls for the cytoplasm and nucleus. **E** Detection of PCNA and γH2AX levels in IPEC-J2s infected with PDCoV. **F** Mutagenesis was performed on HA-PCNA, with Lys residues being substituted with Arg (K168R) and co-transfected with ISG15-overexpression plasmid. **G** N protein expression. **H** The expression of his Max-tagged PCNA or its 168 mutant was observed in cells that utilised the ISG15-conjugating system. NTA resins were used to pull down cell lysates, and then an immunoblot was performed using an anti-Flag antibody. **I** USP18 interacts with PCNA. The expression of Flag-USP18 was induced in IPEC-J2s in the presence of HA-PCNA. Protein A/G magnetic beads were used to immunoprecipitate cell lysates. **J** We overexpressed hisMax-PCNA and ISG15-conjugating system (E1/E2/Flag-ISG15) in cells with and without Flag-USP18. NTA resins were used to pull down cell lysates, and then anti-Flag or anti-Xpress antibodies were used to create an immunoblot. We also directly probed the lysates with the same antibodies (Table 3).

PCNA is a necessary factor in DNA repair and synthesis, and it influences the cell cycle and viral replication. Considering the results of mass spectrometry and other studies, we speculated that PCNA may be involved in ISGylation during PDCoV infection. To evaluate the function of PCNA, we performed PCNA-overexpressed vector transfection and PCNAI1 pretreatment at different concentrations (5, 1, and 0.5 μM), which has been reported as an inhibitor of PCNA by stabilizing the trimer structure of PCNA and reducing PCNA binding to the chromatin [26]. The CCK-8 assay was used to determine the safe concentration of PCNA-I1 (Figure 7A). As shown in Figure 7B and C, PCNA overexpression significantly enhanced PDCoV viral titers and N protein levels. Nevertheless, PCNAI1 inhibited PDCoV replication, suggesting that PCNA plays an important role in PDCoV replication. We also detected the translocation of PCNA from nucleus to the cytoplasm by western blotting. As shown in Figure 7D, PDCoV infection obviously induced expression of PCNA in cytoplasm and reduced expression of PCNA in nucleus. These results indicated that PDCoV acted on PCNA translocation. In addition, γH2AX (another DNA damage marker) and PCNA were tested following PDCoV infection at 12 hpi and 24 hpi (Figure 7E). The infection caused more DNA damage in infected cells than in non-infected cells, as indicated by higher expressions of PCNA and γH2AX. To investigate the influence of ISGylated PCNA on PDCoV replication, we used several approaches to block PCNA ISGylation. First, we constructed a K168R mutant of PCNA, which was indicated as a key site of PCNA ISGylation [6]. As shown in Figure 7F and G, a dramatic decrease in PDCoV replication was observed when ISG15 was overexpressed by additional CMV-ISG15 transfection, and this impact on PDCoV replication could be overturned by complementation of the original PCNA, but not by a non-functional mutant of PCNA. Meanwhile, the ISGylation levels of PCNA WT and its mutant K168R were compared using pulldown (PD) assay proving that K168 is a major ISGylation site in porcine PCNA (Figure 7H). Furthermore, we investigated whether there was an interaction between PCNA and USP18, as demonstrated above in the results for de-ISGylated proteins during PDCoV infection. As shown in (Figure 7I), PCNA could bind to USP18. The ability of PCNA to undergo ISGylation was investigated by overexpressing the ISG15-conjugating system together with HisMax-PCNA in IPEC-J2. NTA resins were then used to pull down the cell lysates. Two bands that were reactive with anti-ISG15 antibodies appeared, and these could eventually be de-ISGylated by co-expression of Flag-USP18 (Figure 7J).

**Table 3:** **Proteins interacting with the ISG15**

Protein name	Function	MW.kDa	Sample	Acc.ID
GBP1	GTPase activity,Molecular; GTP binding	67.7	C	K7GLP8
CDK1	protein kinase activity; ATP binding; protein phosphorylation	31.5	C	A0A8D0J2D2
**IQGAP1**	Signal transduction mechanisms		F,C	A0A4X1UKT5
ITGB (Integrin beta)	Rap1 signaling pathway; PI3K-Akt signaling pathway; Focal adhesion	89.9	F	A0A8D1HI17
**PCNA**	Proliferating cell nuclear antigen	28.1	F,C	A0A4X1UCG8
CCT3	ATP binding	55.8	F,C	A0A8D1KEF5
CCT4	ATP binding	54.8	F,C	A0A5G2QN54
**FLNB (Filamin-B isoform 2)**	protein binding	282.8	F	A0A480YXA1
RAB9B	GTP binding	22.7	F,C	A0A287BH53
RNF(RING-type E3 ubiquitin transferase)	Post-translational modification, protein turnover, chaperones;	53.6	F	A0A8D1DCW0
RAB-11a	Intracellular trafficking, secretion, and vesicular transport	17.5	C	A0A4X1TE59
RAB14	Intracellular trafficking, secretion, and vesicular transport	23.9	F	Q52NJ6
HSP90AB1	RNA processing and modification	94.5	F,C	A0A8D1BLY6
YWHAZ	Cell cycle; Post-translational modification, protein turnover, chaperones	27.7	F,C	A0A480PLY3
HSP90B1	Post-translational modification, protein turnover, chaperones;	92.4	F,C	A0A8D0KEJ4
HSP90AA1	Post-translational modification, protein turnover, chaperones;	90.6	F,C	A0A4X1TS37
TMED10	Intracellular trafficking, secretion, and vesicular transport	24.9	F,C	A0A286ZV95
TMED9	Intracellular trafficking, secretion, and vesicular transport	27.1	F,C	F1S3E0
DDX17	RNA processing and modification	80.2	F,C	A0A8D0V9F7
RPL7	Translation, ribosomal structure and biogenesis;	29.2	F,C	A0A287A8T0
RPL8	Translation, ribosomal structure and biogenesis	24.6	F	K7GNY4
RPL10A	Translation, ribosomal structure and biogenesis	24.8	F	A0A4X1T0H0
RPL12	Translation, ribosomal structure and biogenesis	17.8	F	A0A4X1U700
RPL13	Translation, ribosomal structure and biogenesis	24.3	F	I3LSD3
RPL15	Translation, ribosomal structure and biogenesis	21.9	F	A0A287A452
RPL18	Translation, ribosomal structure and biogenesis	18.9	F	Q0QEV2
RPL23	Translation, ribosomal structure and biogenesis	14.9	F	P62831
VDAC1	voltage-dependent anion channel protein 1	40.6	F	A0A4X1UV26
LAMP1	Lysosomal-associated membrane protein	43.1	F	A0A8D0MH64
HSP60	Post-translational modification, protein turnover, chaperones;	60.9	F	A0A8D0I7D0

Bolded proteins were previously described as direct interacting partners of ISG15

Accession number is the registered number in UniProt database. F: ISG15-overexpressed IPEC-J2 infected with PDCoV. C: Non-infected ISG15-overexpressed IPEC-J2

### HERC5 promotes the anti-viral activity of ISG15 by catalyzing ISGylation of PCNA

**Figure 8:**
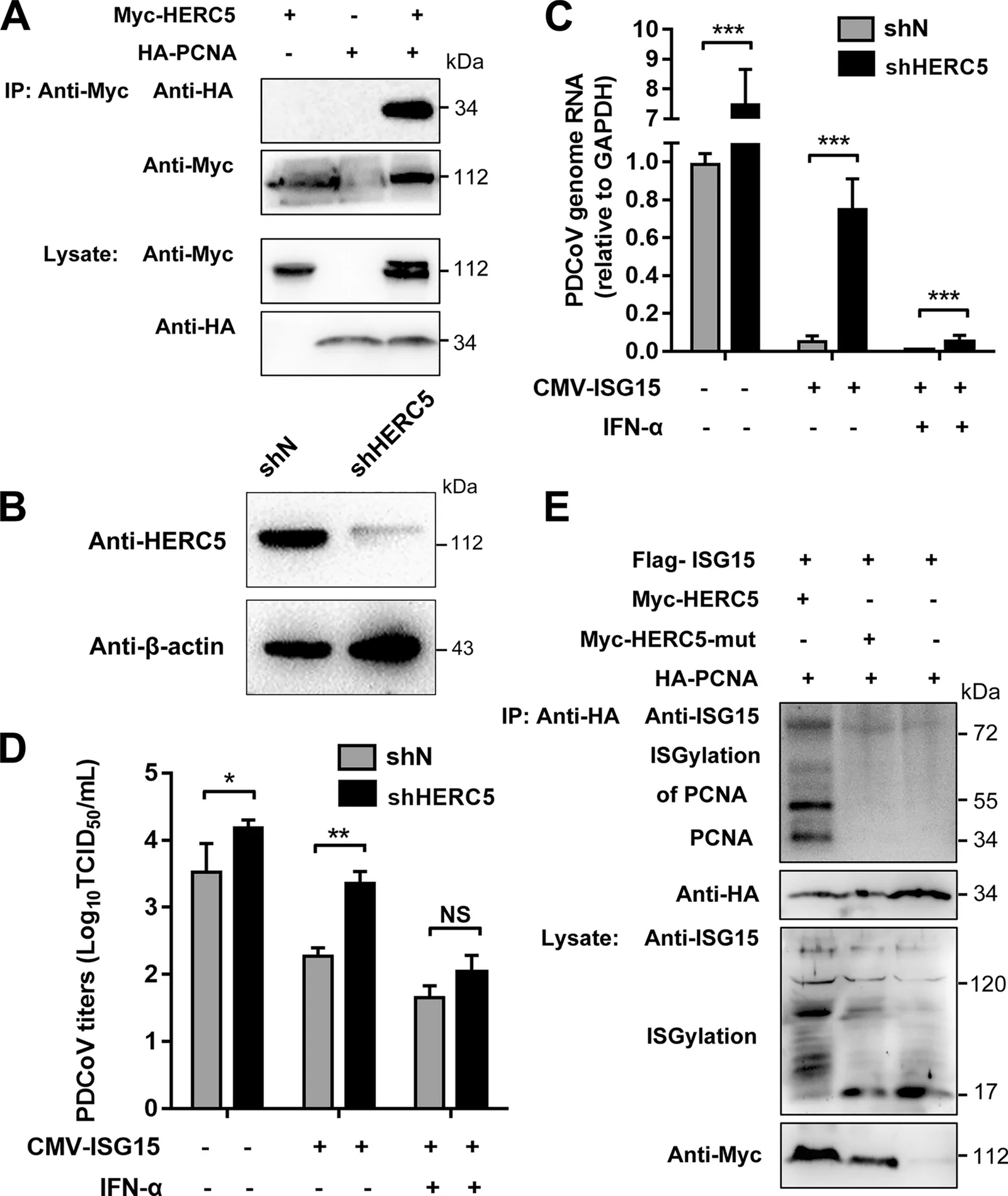
**Interaction between HERC5 and PCNA.** **A** HERC5-Myc was expressed in IFN-α-pretreated IPEC-J2s alongside HA-PCNA. Protein A/G magnetic beads were used to immunoprecipitate cell lysates. **B** Knockdown of HERC5 was evaluated with anti-HERC5 antibody by western blotting. **C** and **D** shN and shHERC5 IPEC-J2s were transfected with Flag-ISG15 or pre-treated with IFN-α followed by PDCoV infection for another 24 h. **D** Cys-994 within the HECT domain of HERC5 was mutated to alanine residue. IPEC-J2s were co-transfected with plasimd expressing Flag–ISG15, HA-PCNA, Ube1L, and UbcH8 with Myc-HERC5 or its mutant.

HERC5 is an ISG15 E3 ligase in swine [4]. To determine whether HERC5 participates in PCNA ISGylation, we tested its binding ability to PCNA. As shown in Figure 8A, there was an obvious interaction between PCNA and HERC5. We used shRNA technology to suppress the expression of HERC5 after IFN-α pretreatment to investigate the effect of HERC5 on ISGylation. The knockdown of HERC5 was evaluated by western blotting Figure 8B IPEC-J2 cells were transfected with either shNegative control (shN) or shHERC5 against HERC5. Following IFN-α pretreatment for 12 h, inducing the ISGylation system and PDCoV incubation for 12 h, the knockdown of HERC5 significantly impaired the inhibition of ISG15-overexpression and IFN-α on PDCoV infection (Figure 8C and D). Since the cysteine 994 residue of the HECT domain in HERC5 is necessary for the activity of ISG15 E3 ligase [23], we constructed an enzymatically dead mutant of HERC5 (C994A) replacing the cysteine 994 residue with an alanine residue via site-specific mutagenesis. As shown in (Figure 8E), ISGylation of PCNA by ISG15 was significantly increased by HERC5 overexpression but not by the HERC5 mutant. Collectively, these results indicate that HERC5 acts as an ISG15 E3 ligase during PDCoV infection that facilitates PCNA ISGylation.

## Discussion

It has been proposed that ISG15 functions as an efficient host factor that protects against viral infections, including influenza A virus [27], SARS-CoV-2 [15], Peste des petits ruminants virus [28], and classical swine fever virus [8]. This present work demonstrated the widespread expression of ISG15 in swine cells (see Figure 2A) and provided compelling evidence that ISG15 plays a significant role in counteracting PDCoV infection in IPEC-J2 cells via overexpression plasmid transfection. In contrast, knockout of ISG15 mediated by CRISPR-Cas9 enhances PDCoV replication (Figure 2 and 3).

This phenomenon motivated us to investigate whether ISGylation is involved in the inhibition of ISG15 in PDCoVs, a process that remains incompletely understood. We demonstrated that ISG15 anti-viral activity against PDCoV infection requires protein ISGylation through a conjugation-defective ISG15 mutant which is typically used to incapacitate ISG15 normal ISGylation [29]. The results illustrated that key site mutations reduced the anti-viral activity of ISG15 against PDCoV, suggesting that ISGylation plays a necessary role in ISG15 anti-viral activity (Figure 4).

The vast majority of these potentially new ISGylation target proteins have not yet been validated in swine cells. Intriguingly, we found that only overexpressed ISG15 could induce ISGylation without co-translating with other ISGylation enzymes or IFN pretreatment. Thus, we constructed a stable ISG15-overexpressing swine cell line to gain a greater understanding of the manner in which ISG15 induces cellular machinery in the context of PDCoV infection (Figure 5).

Using TAP-LS/analysis, IQGAP1, PCNA, and filamin-B were found to be ISG15 interact candidatesin our study, and they have been previously reported to interact with ISG15. ISGylation of IQGAP1 may be associated with the regulation of the cytoskeleton in breast cancer cells [30]. The modification of filamin B by ISG15 functions as a regulatory gate that acts as a negative feedback mechanism for the desensitisation of the type I IFN pathway. [31].

PCNA has been demonstrated to function as a scaffold, thereby facilitating the recruitment of DNA repair and chromatin remodelling proteins, which are essential for the replication of DNA. Inhibitors of PCNA impede the recruitment of inhibiting viral DNA synthesis and coupled processes [32]. We selected PCNA for further investigation, aiming to determine whether it could serve as a target protein for ISGylation and play a role in PDCoV infection. We first proved that PCNA contributed to PDCoV replication. Other studies have shown that PCNA consistently promotes viral multiplication, including HBV, EBV [33], PRRSV, and HSV-1 [32]. SARS-CoV-2 M protein interacted with PCNA to promote replication [18]. Consistently, in our study, we documented a reallocation of PCNA from the nucleus to the cytoplasm and the increase of PCNA and γH2AX (another DNA damage marker) expression. But we found that there is no interaction between PCNA and PDCoV M protein. During HSV-1 infection, inhibitors of cellular PCNA block the recruitment of key viral and cellular factors to viral DNA, thereby inhibiting viral DNA synthesis and associated processes[32].

Next, we investigated how PCNA ISGylation affects PDCoV replication. We found that PCNA specifically interacted with USP18 (Figure 7I). Overexpression of USP18 was sufficient to sustain PCNA de-ISGylation, suggesting that USP18 is responsible for the de-ISGylation of PCNA. In our previous study, we expressed three kinds of ISG15 E3 ligases HERC5 (GenBank: OR493513.1; SUS), HERC6 (GenBank: MZ826134.1; SUS), EFP (GenBank: D21205.1 HOMO). Protein interaction between three kinds of E3 ligases and PCNA were performed to find only HERC5 bound to PCNA. In the context of coronavirus (and other RNA virus) infections, ISGylation can confer antiviral activity by promoting virus sensing (for example, by promoting MDA5 or STING activation), or by blocking coronavirus/SARS-CoV-2 N function through direct ISGylation by HERC5 [34–36]. HERC5 was identified as an essential E3 ligase that regulates SARS-CoV-2 nsp8 stability through ISGylation [37]. We emphasize here that the facilitation effects of HERC5 on ISGyalted PCNA during PDCoV infection are specific because they were abolished as a result of mutations in the cysteine 994 residue of HERC5, which specifically prevented ISG15/PCNA binding. We speculate that de-ISGylation of PCNA by USP18 acts as a safeguard against overactive ISGylation. Therefore, the levels of PCNA ISGylation can be positively regulated through the E3 ligase HERC5 or negatively regulated by USP18 activities.

## Supplementary Information

 **Additional file 1 Proteins associated with and/or modified by ISG15 are to be analyzed.** (A) Venn diagram showing the number of common or different proteins identified among all interacting proteins from the CMV-ISG15 (A1) and CMV-ISG15+PDCoV (A2) groups. (B) Venn diagram illustrating the GO, KEGG, COG, and IPR databases utilised for the annotation of results. (C) Comparisom of two-step purification. The samples are shown in lanes 1, 2 and 3. These are total cell lysates of one-step samples purified by Flag tag, one-step samples purified by His tag and two-step samples purified by His tag, respectively. The samples in lanes 4, 5 and 6 are total cell lysates of one-step samples purified by Flag tag, one-step samples purified by His tag and two-step samples purified by His tag, respectively. The samples in these last three lanes are from PDCoV-infected CMV-ISG15. The white circles represent Flag-His-ISG15 fusion protein. (D) Pathway analysis of the identified interacting partners with ISG15 in tow compared groups. (E) IPR annotation from the CMV-ISG15 with PDCoV infection.

## Data Availability

The Data generated during the study is available at Mendeley Data at http://doi.org/10.17632/v3n4nz7ffz.1. The mass spectrometry proteomics data have been deposited in the Proteome Xchange Consortium (http://proteomecentral.proteomexchange.org) via the iProX partner repository with the dataset identifier PXD062294.
