# Supplementary material for: Modification of PCNA by ISG15 plays a crucial role in porcine deltacoronavirus infection
Source: Vet Res. 2026 Jul 11;57:134. doi: 10.1186/s13567-026-01802-1 (PMC13356795; doi:10.1186/s13567-026-01802-1)
Supplement: Supplementary file 1 — Additional file 1 Proteins associated with and/or modified by ISG15 are to be analyzed. (A) Venn diagram showing the number of common or different proteins identified among all interacting proteins from the CMV-ISG15 (A1) and CMV-ISG15+PDCoV (A2) groups. (B) Venn diagram illustrating the GO, KEGG, COG, and IPR databases utilised for the annotation of results. (C) Comparisom of two-step purification. The samples are shown in lanes 1, 2 and 3. These are total cell lysates of one-step samples purified by Flag tag, one-step samples purified by His tag and two-step samples purified by His tag, respectively. The samples in lanes 4, 5 and 6 are total cell lysates of one-step samples purified by Flag tag, one-step samples purified by His tag and two-step samples purified by His tag, respectively. The samples in these last three lanes are from PDCoV-infected CMV-ISG15. The white circles represent Flag-His-ISG15 fusion protein. (D) Pathway analysis of the identified interacting partners with ISG15 in tow compared groups. (E) IPR annotation from the CMV-ISG15 with PDCoV infection. [file 13567_2026_1802_MOESM1_ESM.docx]

**Additional file 1**


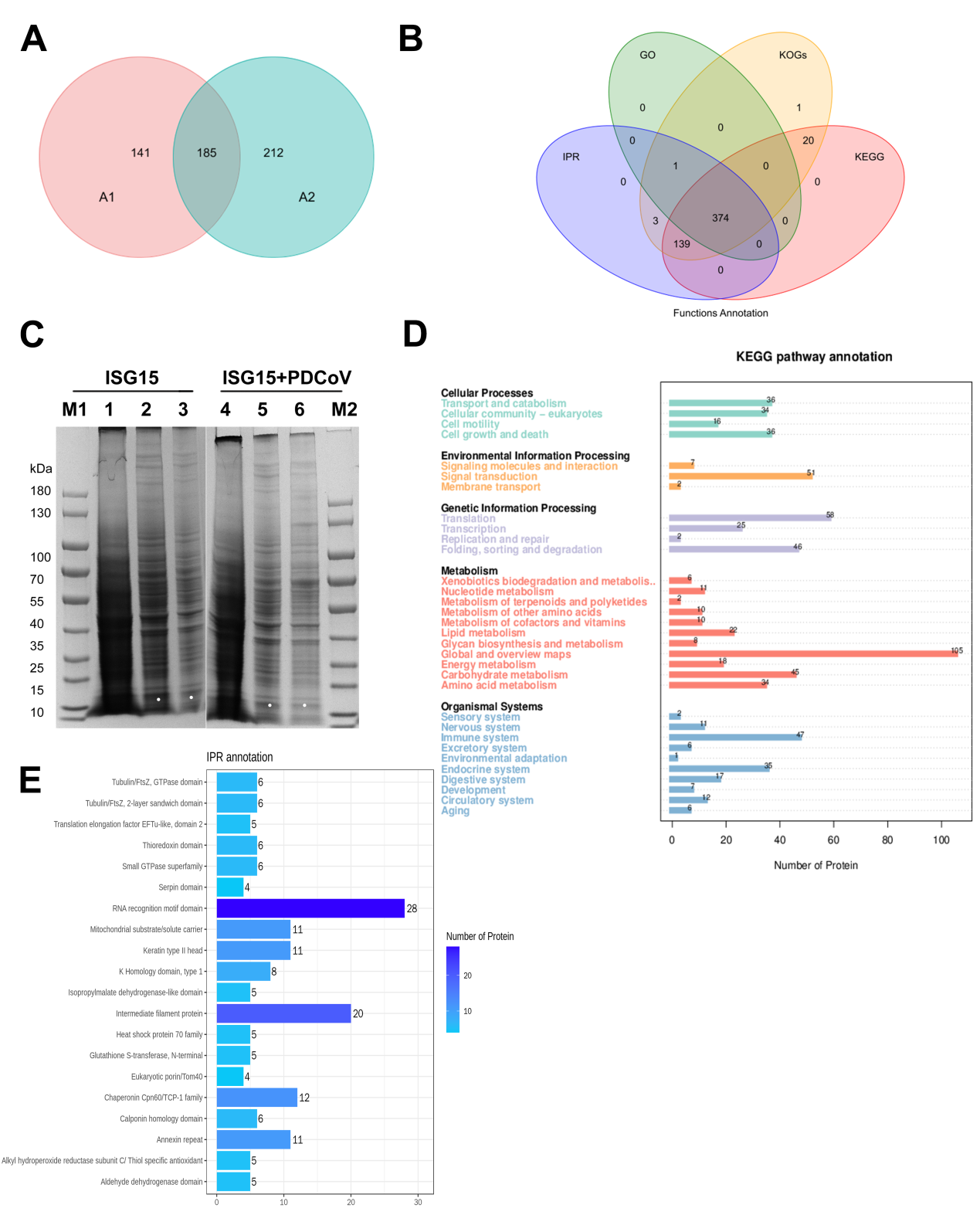


**Additional file 1** (A) Venn diagram of all interacting proteins from the CMV-ISG15 (A1) and CMV-ISG15+PDCoV (A2) groups identified representing the number of their common or different proteins. (B) Venn diagram of the GO, KEGG, COG, and IPR databases used to annotate the results. (C) Comparisom of two-step purification by SDS-PAGE gel with coomassie brilliant blue stained. Total cells lysates (lane 1) one-step samples purified by Flag tag (lane 2) and two-step samples purified by His tag (lane 3) of CMV-ISG15 and total cells lysates (lane 4) one-step samples purified by Flag tag (lane 5) and two-step samples purified by His tag (lane 6) of PDCoV-infected CMV-ISG15 are shown. The white circles represent Flag-His-ISG15 fusion protein. (D) Pathway analysis of the identified interacting partners with ISG15 in tow compared groups. (E) IPR annotation from the CMV-ISG15 with PDCoV infection.
